# Frailty and risks of all-cause and cause-specific death in community-dwelling adults: a systematic review and meta-analysis

**DOI:** 10.1186/s12877-022-03404-w

**Published:** 2022-09-02

**Authors:** Yang Peng, Guo-Chao Zhong, Xiaoli Zhou, Lijuan Guan, Lihua Zhou

**Affiliations:** 1 Geriatric Diseases Institute of Chengdu, Department of Geriatrics, Chengdu Fifth People’s Hospital, Chengdu, 611137 China; 2 The Second Clinical Medical College, Affiliated Fifth People’s Hospital of Chengdu University of Traditional Chinese Medicine, Chengdu, 611137 China; 3 Department of Hepatobiliary Surgery, the Second Affiliated Hospital of Chongqing Medical University, Chongqing, China; 4 Department of Laboratory Medicine, Sichuan Provincial People’s Hospital, University of Electronic Science and Technology of China, Chengdu, China; 5 Chinese Academy of Sciences Sichuan Translational Medicine Research Hospital, Chengdu, China

**Keywords:** Frailty, All-cause mortality, Cause-specific mortality, meta-analysis, Cardiovascular disease, cancer, Respiratory illness

## Abstract

**Background:**

The associations of frailty with all-cause and cause-specific mortality remain unclear. Therefore, we performed this meta-analysis to fill this gap.

**Methods:**

We searched the PubMed and Embase databases through June 2022. Prospective cohort studies or clinical trials examining frailty were evaluated, and the multiple adjusted risk estimates of all-cause and cause-specific mortality, such as death from cardiovascular disease (CVD), cancer, respiratory illness, dementia, infection, and coronavirus disease 2019 (COVID-19), were included. A random effects model was used to calculate the summary hazard ratio (HR).

**Results:**

Fifty-eight studies were included for the qualitative systematic review, of which fifty-six studies were eligible for the quantitative meta-analysis, and the studies included a total of 1,852,951 individuals and more than 145,276 deaths. Compared with healthy adults, frail adults had a significantly higher risk of mortality from all causes (HR 2.40; 95% CI 2.17–2.65), CVD (HR 2.64; 95% CI 2.20–3.17), respiratory illness (HR 4.91; 95% CI 2.97–8.12), and cancer (HR 1.97; 95% CI 1.50–2.57). Similar results were found for the association between prefrail adults and mortality risk. In addition, based on the studies that have reported the HRs of the mortality risk per 0.1 and per 0.01 increase in the frailty index, we obtained consistent results.

**Conclusions:**

The present study demonstrated that frailty was not only significantly related to an increased risk of all-cause mortality but was also a strong predictor of cause-specific mortality from CVD, cancer, and respiratory illness in community-dwelling adults. More studies are warranted to clarify the relationship between frailty and cause-specific mortality from dementia, infection, and COVID-19.

**Trial registration:**

PROSPERO (CRD42021276021).

**Supplementary Information:**

The online version contains supplementary material available at 10.1186/s12877-022-03404-w.

## Background

Global ageing results in extensive concerns about various geriatric syndromes [1]. As one of the most common geriatric syndromes, frailty is a condition of an attenuated physiological reserve, which is characterized by an impaired response and an increased vulnerability to stressor events [2]. Considering that frailty is a frequently used clinical indicator of functional ageing, the prevalence of frailty varies from 4.0 to 59.1% in community-dwelling older people [3].

Based on the different theories, various tools have been established to assess frailty status. One of the most popular assessment tools is the frailty phenotype (FP), which categorizes the population into frail, prefrail, and robust or not frail according to five criteria (unintentional weight loss, self-reported exhaustion, low energy expenditure, a slow gait speed, and a weak grip strength) [4]. Another common tool is the frailty index (FI), which is measured as the proportion of accumulated deficits and defines frailty by predefined cut-points [5]. The FRAIL scale (FS) is also a widely used frailty screening tool that can recognize either a frail or a prefrail status quickly in terms of five self-reported items: fatigue, resistance, ambulation, illnesses, and loss of weight [6]. Despite conceptual differences, these tools have all been well validated in subsequent studies and have been widely used in clinical and scientific research [7].

Many studies have explored the association between frailty and various adverse health outcomes, such as falls, fractures, disabilities, institutionalization, hospitalization, and death, in the general population, especially in older adults [8–10]. To date, numerous studies have reached a consensus that frailty is a predictor of mortality. However, these studies were usually limited to mortality in specific populations, such as perioperative patients, [11] nursing home residents, [12] and patients with diseases such as tumours, [13] heart failure, [14] coronavirus disease 2019 (COVID-19), [15] etc. Furthermore, owing to the relatively small sample sizes used to evaluate cause-specific mortality in previous research, only all-cause mortality was regarded as the endpoint in most relevant systematic reviews, meta-analyses and umbrella reviews [16–22]. Although a 2017 meta-analysis investigated the associations of frailty with morbidity and mortality from cardiovascular disease (CVD), only 2 included studies provided data on CVD-related mortality, and these data were limited to adults older than 65 and survivors after an acute coronary syndrome [23].

An ageing society is associated with a higher risk of frailty and prefrailty in the community population [24]. In this context, emerging community-based studies have provided more evidence of frailty and all-cause mortality and cause-specific deaths from CVD, cancer, respiratory illness, dementia, infection, COVID-19, etc. [25–28] Nonetheless, the conclusion is still ambiguous. Previous meta-analyses mainly focused on single frailty assessment tools [16–18], and there is no systematic review and meta-analysis on the relationship between frailty status and cause-specific mortality thus far. Therefore, to quantify the associations of frailty status with all-cause and cause-specific mortality, we performed this meta-analysis.

## Methods

We performed this systematic review and meta-analysis following the Preferred Reporting Items for Systematic Reviews and Meta-analyses (PRISMA) guidelines [29]. No ethics committee approval was required for this study. The study protocol was registered at PROSPERO (CRD42021276021) [30].

### Literature search

We performed an electronic literature search of the PubMed and Embase databases from inception to August 2021 to identify the relevant studies using a combination of terms: “frailty” or “frail” and “mortality” or “death”. An updated literature search was performed in June 2022. The language was restricted to English. A detailed description of the search strategy is supplemented in Supplementary Table 1. We manually checked the references of pertinent articles for additional studies and contacted the original author when necessary.

### Study selection

Two of the authors (Y.P. and G.C.Z.) independently screened the citations in accordance with the preset inclusion and exclusion criteria. Any disagreements were settled by consulting a third reviewer (L.Z.).

The included studies were required to fulfil the following criteria: 1) prospective cohort studies or clinical trials reporting all-cause or cause-specific mortality, such as deaths from CVD, cancer, respiratory illness, dementia, infection, and COVID-19; 2) the study participants were adults over the age of 18 in community-dwelling settings; 3) frailty status was defined by one of the three most commonly used tools (i.e., the FP, FI, or FS); 4) the multiple adjusted risk estimates with corresponding 95% confidence intervals (CIs) were available; and 5) when the same cohort was used in multiple publications, the latest published one with the largest number of events was included.

Studies were excluded if they 1) defined frailty status by other evaluation methods; 2) investigated the association of changes in frailty status or the combined impact of other factors with mortality; 3) focused on non-community participants, such as those in hospitals, nursing homes or patients with certain diseases; and 4) were conference abstracts, cross-sectional analyses, review articles, editorials, letters, or published errata.

### Data extraction and quality assessment

Two investigators (Y.P. and G.C.Z.) independently extracted the data and evaluated the methodological quality of the selected studies. Disagreements were resolved by discussion with a third author (L.Z.). Using a predesigned data extraction form, the following information was recorded: the name of the first author, publication year, study location, mean age, follow-up years, sample size, sex, assessment tools, status of frailty, cause and number of deaths, outcome assessment, fully adjusted risk estimate and the corresponding 95% CIs, and adjustment factors. Methodological quality was evaluated through the Newcastle–Ottawa Quality Assessment Scale (NOS) [31]. Studies with a score of 6 or more points were deemed to be of high quality, and the maximum score was 9 for each study.

### Statistical analysis

In our study, the hazard ratio (HR) was used as a common measure to estimate the combined effect size, and the odds ratio (OR) was regarded as equivalent. We conducted a random effects meta-analysis when more than 3 studies provided the same effect measure for all-cause or certain cause-specific mortality in terms of the following categories: robust, prefrail, and frail status. For some studies [32–36] that provided HRs categorized by sex, age range or severity of frailty status, we combined the HRs through a random effects model to yield a summary HR.

Since some studies provided HRs per 0.1 or 0.01 increase in the FI, we also combined the HRs for mortality per 0.1 or 0.01 FI increment. For one study [27] that evaluated the mortality from ischaemic heart disease and cerebrovascular disease and another study [28] that evaluated the mortality from stroke, heart attack, and other CVDs separately, we combined the HRs through a random effects model to yield a summary HR for the mortality from CVD, since these diseases are important components of CVD. Circulatory diseases, heart disease, and CVD were regarded as equivalent diseases in our analysis. Likewise, neoplasms were deemed as cancer.

The Q statistic (significance set at *P* < 0.10) and I^2^ statistic (I^2^ > 75.0%, 50.0–75.0, and < 50.0% signified substantial, moderate, and low heterogeneity, respectively) were adopted to quantify the heterogeneity across the studies. To clarify the potential source of heterogeneity, we conducted sensitivity analyses by omitting one study in turn, repeating the meta-analysis through a fixed effects model, and changing the eligibility criteria. It is worth noting that the sensitivity analyses were conducted only for all-cause and CVD mortality due to the limited number of studies that evaluated cancer and respiratory illness mortality.

We selected Begg’s and Egger’s tests to determine if there was publication bias in our meta-analysis. STATA software (version 15.0, StataCorp LP, College Station, Texas, USA) was used for all statistical analyses, and the statistical significance level was defined as *P* < 0.05 under a two-sided test.

## Results

### Search results

**Fig. 1:**
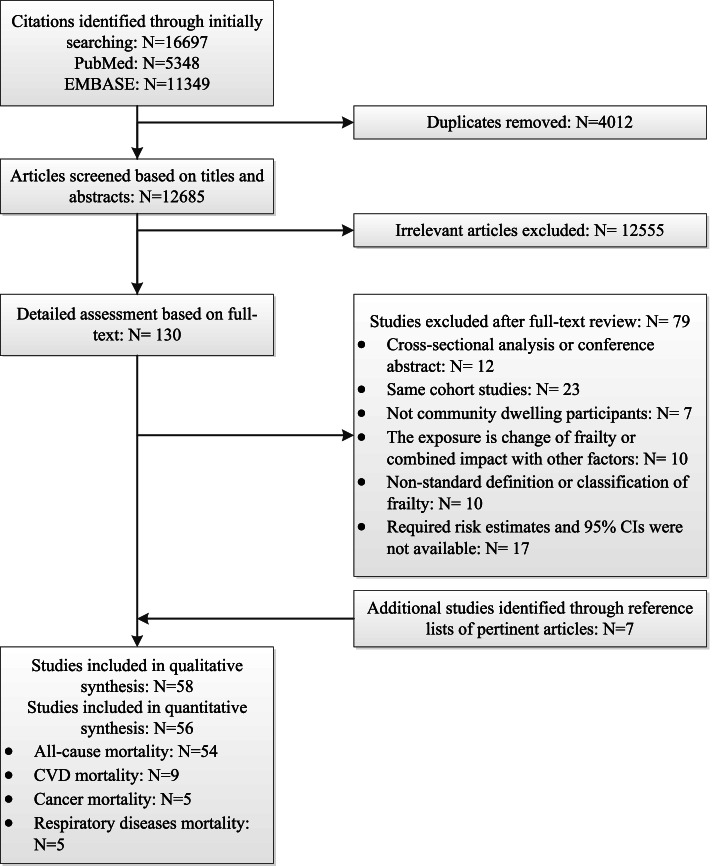
Flowchart of the included studies

Through an initial systematic search, a total of 16,697 citations were identified from the databases, of which 12,685 citations remained after the removal of duplicate studies. After reviewing the titles and abstracts, 12,555 irrelevant articles were excluded. A total of 130 studies remained for further full-text assessment, and a list of studies excluded after a detailed assessment based on full text are presented in Supplementary Table 2. In addition, 7 additional studies were found through reference lists of pertinent articles. Finally, 58 studies were included for the qualitative systematic review, and 56 studies were eligible for the quantitative meta-analysis. The detailed selection process and reasons for exclusion are shown in the flowchart (Fig. 1).

### Study characteristics and quality assessment

The main characteristics of the included studies are summarized in Table 1. The 58 studies included 1,852,951 individuals and more than 145,276 deaths, involving all-cause death and specific causes of death, including CVD, cancer, respiratory illness, dementia, infection, and COVID-19. The death-related information was available from sources such as the death registry, death certificates, National Death Index, structured interview, and standard report. The study locations were spread all around the world. Most of the included studies were prospective cohort studies, apart from the study by Farooqi et al., [37] which was a pooled analysis of prospective clinical trials. The follow-up duration ranged from 0.6 to 30 years. The mean age of the baseline population varied from 44·0 to 93.7 years. In addition to six studies [38–43] that only enrolled male or female individuals, the other studies all consisted of both sexes. Almost all the identified studies provided corresponding HRs for the risk of death, with potential adjustment factors including age and sex, but three studies [25, 44, 45] reported the OR.

**Table 1:** The characteristics of the included studies

Study	Study location	Follow-up, years	Mean age, years	Sample size	Deaths	Frailty assessment	Outcome assessment	Adjustment factors
Mak et al., 2021 [25]	U. K.	0.6	67.6	T 410199 W 226018 M 184181	All-cause 3186 COVID-19514	**FP**: Frail Prefrail Non-frail **FI**: Frail (> 0.21) Least fit (0.1–0.21) Less fit (0.03–0.1) Relatively fit (≤0.03)	Death registry	age, sex
Gilmour et al., 2021 [35]	Canada	3–5	74	T 29302 W 16724 M 12578	All-cause 3540 CVD NA Cancer NA Respiratory illness NA	**FI**: Most frail (≥ 0.45) Moderately frail (0.21–0.45) Prefrail (0.10–0.21) Robust (≤ 0.10)	Vital Statistics Database	age, sex, alcohol use in the past 12 months (regular, occasional, never), smoking (current, former, never), household education, marital status, living arrangements
Lohman et al., 2020 [26]	U. S.	5.9	73.8	T 10490	All-cause 2148 CVD 738 Dementia 131 Cancer 490 Respiratory illness 265	**FP**: Frail Prefrail Non-frail	National Death Index	age, race, sex, years of education, marital status, cognitive score, smoking status, number of chronic health conditions, number of ADL limitations, history of heart disease, cancer, diabetes, respiratory disease, or cerebrovascular disease/stroke
Hoogendijk et al., 2020 [46]	Italy	6	75.2	T 1129 W 642 M 487	All-cause 267 CVD 128	**FI**:0.01	Death registry	age, sex, partner status, educational level, smoking
Farooqi et al., 2020 [37]	Canada	3.2	70.8	T 154696 W 57238 M 97548	All-cause 15,067 CVD 9432	**FI**: Frail (> 0.21) Prefrail (0.1–0.21) Non-frail (≤0.1)	Standard case report forms	age, sex, ethnicity, smoking history, history of myocardial infarction, stroke, heart failure, diabetes mellitus, hypertension, peripheral arterial disease, elevated BMI, high cholesterol
Fan et al., 2020 [27]	China	10·8	52·0	T 512723 W 302521 M 210202	All-cause 49,371 CVD 18421 Cancer 15,750 Infection 629 Respiratory illness 4652	**FI**: Frail (≥ 0.25) Prefrail (0.1–0.25) Robust (≤0.1)	Death registry	age, education level, tobacco smoking, alcohol, intake frequency of fresh fruits, vegetables, red meat, family disease history of heart attack, stroke, cancer
Li et al., 2019 [34]	Sweden	17–20	<60	T 42953 W 23029 M 19924	All-cause 12,222 CVD 3270 Cancer 3302 Respiratory illness 1051	**FI**:0.1	Death registry	attained age as time scale, BMI, years of education, tobacco use status, history of CVD, respiratory diseases, cancer
Grabovac et al., 2019 [28]	Europe	1–12	64.2	T 24634 W 11435 M 13199	All-cause 2557 CVD 905 Cancer 770 Respiratory illness 146 Infection 140	**FI**: men: Frail (> 3.005) Prefrail (1.211–3.005) Robust (< 1.211) **FI**: women: Frail (> 2.130) Prefrail (0.315–2.130) Robust (< 0.315)	Interview with a proxy (family or household member)	sex, age, education, BMI, smoking, alcohol consumption, numbers of comorbidities
Yuki et al., 2018 [47]	Japan	M:7.7 W:7.9	>70	T 841 W 434 M 407	All-cause 113 CVD 30 Cancer 45	**FP**: Frail Prefrail Non-frail	Death registry	age, sex, percentage body fat, education, total physical activity, total caloric intake, alcohol intake, current smoking status, household income, Epidemiologic Studies Depression Scale score, MMSE score, number of comorbidities
Higueras-Fresnillo et al., 2018 [48]	Spain	14	>70	T 3896	All-cause 1801 CVD 672	**FS**: Frail Prefrail Non-frail	National Death Index	age, sex, educational level, smoking status, alcohol consumption, BMI, waist circumference, MMSE score
Crow et al., 2018 [49]	U. S.	7.98	71.1	T 4984 W 2531 M 2453	All-cause 1901 CVD 521	**FP**: Frail Prefrail Non-frail	National Death Index	age, sex, race, education, smoking, diabetes, heart failure, cancer, coronary artery disease, arthritis
Adabag et al., 2018 [38]	U. S.	9.2	76.4	M 3135	All-cause 1275 CVD 445	**FP**: Frail Intermediate stage Robust	Death certificates/ medical records	site, age, race, smoking and comorbid medical conditions (stroke, diabetes mellitus, hypertension, coronary heart disease, peripheral vascular disease, valvular heart disease, CHF, COPD)
Jiang et al., 2017 [50]	Sweden	30	63.2	T 1477 W 854 M 623	All-cause 975 CVD 347 Cancer 232 Dementia 78	**FI**: Frail (> 0.21) Least fit (0.1–0.21) Less fit (0.03–0.1) Relatively fit (≤0.03)	Death registry	age, smoking status
Hou et al.,2022 [32]	U.K.	11.23	56.28	T 449971 W 250354 M 199617	All-cause 23,163	**FS**: Frail Prefrail Non-frail	Death registry	age, TDI, income, ethnicity, education level, employment status, smoking, alcohol consumption, healthy diet score, BMI, cholesterol, CRP, HDL, LDL, triglycerides, HbA1c
Baek et al.,2022 [51]	Korea	12	61.7	T 10254 W 5791 M 4463	All-cause 2196	**FI**: Frail (≥0.25) Pre-frail (0.1–0.25) Robust (≤0.1)	Interview with participants	age, marital status, education level, labor status, household income, cigarette smoking, alcohol drinking
Zhang et al.,2021 [15, 52]	China	13	74.74	T 1459 W 750 M 709	All-cause 938	**FI**: Frail Non-frail	Interview with a proxy (family or neighborhood)	age, sex, chronic diseases (hypertension, diabetes, heart disease, COPD)
Wang et al.,2021 [53]	China	4.58	85.8	T 13859 W 7607 M 6252	All-cause NA	**FP**: Frail Prefrail Robust	Structured interview	education, household income, smoke status, comorbidity count at baseline
Shi et al.,2021 [54]	China	11	72.05	T 1246 W 727 M 519	All-cause 476	**FI:**0.01	Standard forms	age, gender, years of education, marital status, employment status
Barker et al.,2021 [55]	South Africa	1.42	61.3	T 3989 W 2175 M 1814	All-cause 135	**FI:**0.01	Interview with family	age, sex
Lee et al.,2021 [44]	Korea	3	74.6	T 1292 W 717 M 575	All-cause NA	**FS**: Frail Prefrail Non-frail	Structured interview	age, gender
Castellana et al.,2021 [45]	Italy	4.64	73.55	T 1929 W 955 M 974	All-cause NA	**FP**: Frail Prefrail Robust	Death registry	age, sex, education, multimorbidity
Wuorela et al.,2020 [56]	Finland	27	70	T 962 W NA M NA	All-cause NA	**FI:** Frail (≥0.25) Pre-frail (0.9–0.24) Robust (≤0.08)	Death registry	gender
Salminen et al.,2020 [57]	Finland	18	72.7	T 1152 W 657 M 495	All-cause 776	**FS**: Frail Prefrail Non-frail	Death registry	age, gender
Dallmeier et al.,2020 [33]	Germany	6	74	T 1204 W 692 M 512	All-cause 196	**FI:**0.1	Death registry	age, years of school education, smoking, alcohol intake
Wang et al.,2019 [58]	Taiwan	6.62	74	T 921 W 443 M 478	All-cause 161	**FP**: Frail Prefrail Robust	National Death Database	age, sex, education, marital status, BMI, smoking, alcohol drinking, physical activity, exercising program, hypertension, diabetes mellitus, heart disease, hyperlipidemia, gout, hyperuricemia, arthritis, osteoporosis, stroke, cataract, fall history, sleep impairment, cognitive function
Shi et al.,2019 [59]	China	3	75.4	T 1788 W 958 M 830	All-cause 149	**FP**: Frail Prefrail Robust **FI:** Frail (>0.21) Pre-frail (0.1–0.21) Robust (≤0.1)	Death registry	age, gender, marital status, education level, smoking status, drinking status, BMI, hypertension, diabetes, mild cognitive impairment
Keeble et al.,2019 [60]	U.K.	7	85	T 726 W NA M NA	All-cause NA	**FI:** Frail ≥0.25 Non-frail<0.25	Death registry	gender
Jacobsen et al.,2019 [61]	Denmark	1.13	NA	T 7327 W 3829 M 3498	All-cause 49	**FP**: Frail Prefrail Robust	Death registry	age, sex
Zucchelli et al.,2018 [62]	Sweden	13.2	67.1	T 1115 W 642 M 473	All-cause 263	**FP**: Frail Prefrail Non-frail	Death registry	age, sex, education, cardio-metabolic-, neuro-psychiatric-, musculo-skeletal diseases, cognitive deficit, high CRP, malnutrition
Lee et al.,2018 [63]	Korea	3	72.9	T 11266 W 6726 M 4540	All-cause 738	**FP**: Frail Prefrail Non-frail	Interview with the surviving spouse	age, gender, marital status, education, household income, smoking, alcohol drinking, self-rated health, comorbidity, depressive symptoms
Langholz et al.,2018 [64]	Norway	10.1	77.4	T 712 W 367 M 345	All-cause 501	**FP**: Frail Prefrail Non-frail	Death registry	age, comorbidity, disability, smoking, education
Schoufour et al.,2017 [65]	Netherlands	9.5	65.7	T 11539 W 6677 M 4862	All-cause 3902	**FI:**0.01	Death registry	age, gender, cohort
Pereira et al.,2017 [66]	Brazil	5	72.1	T 689 W 474 M 215	All-cause 56	**FI:** Frail (≥ 0.25) Pre-frail (0.1–0.25) Robust (≤0.1)	Mortality Information System	age, gender
Papachristou et al.,2017 [39]	U.K.	2.96	77.95	M 1198	All-cause 83	**FP**: Frail Prefrail Non-frail	Death certificates	age
Hoogendijk et al.,2017 [67]	Netherlands	19	NA	T 2218 W NA M NA	All-cause 1520	**FI:**0.01	Death registry	age, sex
Turusheva et al.,2016 [68]	Russia	5	79	T 306 W 233 M 73	All-cause 120	**FP**: Frail Prefrail Robust	Official reports	age, sex, number of comorbidities at the individual level
Lin et al.,2016 [69]	Taiwan	4.3	66	T 1245 W 566 M 679	All-cause 139	**FI:** Frail ≥0.2 Non-frail<0.2 FI:0.01	Death registry	age, sex
Hyde et al.,2016 [70]	Australia	6.8	60.7	T 363 W 198 M 165	All-cause NA	**FI:** Frail ≥0.2 Non-frail<0.2	Death Database	age, sex, education, alcohol use, smoking, chewing tobacco
Díaz de León González et al.,2016 [71]	Mexico	2.4	66.9	T 4729 W 2527 M 2202	All-cause 212	**FS**: Frail Prefrail Robust	NA	age, sex, number of depressive symptoms, cognitive score and help in at least one ADL
Bartley et al.,2016 [36]	U.S.	6.5	78.5	T 2356 W 1174 M 1182	All-cause 500	**FI:** Frailest (> 0.30) Frail (0.21–0.30) At risk (0.11–0.20) Fit (≤0.10)	NA	age, education, sex
Jotheeswaran et al.,2015 [72]	Seven LMICs^a^	3.9	74.1	T 13924 W 7703 M 6221	All-cause 2306	**FP**: Frail Prefrail Non-frail	Verbal autopsy interview	age, sex, education, disability, health conditions (dementia, depression, number of physical impairments, stroke)
Kulmala et al.,2014 [73]	Finland	4	82.1	T 654 W 455 M 199	All-cause 173	**FP**: Frail Prefrail Robust	Death registry	age, group (intervention vs control), education, smoking, Functional Comorbidity Index, functional capacity (Barthel Index), number of medicines
Ravindrarajah et al.,2013 [40]	Europe	4.3	59.9	M 2929	All-cause 193	**FP/FS**: Frail Prefrail Robust **FI:** Frail (>0.21) Pre-frail (0.13–0.21) Robust(<0.13)	Interview with the relatives	age, center, smoking status, partner status
Garre-Olmo et al.,2013 [74]	Spain	3.6	81.7	T 875 W 509 M 366	All-cause 52	**FI:** Frail ≥0.5 Non-frail<0.5	Interview	age, sex, civil status, the physical, mental, and social frailty phenotype
Abizanda et al.,2013 [75]	Spain	1.46	79.4	T 993 W 601 M 392	All-cause 105	**FP**: Frail Prefrail Non-frail	Telephone interview and Death registry	age, sex, Barthel Index, Charlson Index
Rockwood et al.,2011 [76, 77]	Canada	12	44	T 14713 W 7974 M 6739	All-cause 2020	**FI:** Frail (> 0.21) Least fit (0.10–0.21) Less fit (0.03–0.10) Relatively fit (≤ 0.03) **FI:**0.01	Death certificate	age, sex, education level
Graham et al.,2009 [78]	U.S.	10	74.5	T 1996 W 1168 M 828	All-cause 892	**FP**: Frail Prefrail Non-frail	National Death Index	age, gender, marital status, BMI, smoking status, heart attack, stroke, hypertension, cancer, hip fracture, diabetes, ADL and IADL limitations, cognitive function, depressive symptoms, self-rated health
Avila-Funes et al.,2008 [85]	France	4	74.1	T 6078 W 3724 M 2354	All-cause 316	**FP**: Frail Prefrail Non-frail	Interviews with family/Medical records	sex, education level, income, smoking status, alcohol use, number of chronic diseases, self-reported health, Epidemiologic Studies-Depression scale score, MMSE, baseline disability (mobility, IADL, ADL)
Ensrud et al.,2007 [41]	U.S.	9.2	76.7	W 6724	All-cause 2520	**FP**: Frail Prefrail Robust	Death certificate	age, health status, smoking, estrogen use, education, history of fracture, selected medical conditions (stroke, diabetes, hypertension, parkinsonism, dementia, coronary heart disease, COPD, nonskin cancer, fall history, depressive symptoms), cognitive function, functional status, BMI, femoral neck bone mineral density
Woods et al.,2005 [42]	U.S.	5.9	NA	W 40657	All-cause 2497	**FP**: Frail Prefrail Non-frail	Medical records	age, income, education, ethnicity, health risk variables (BMI, smoking, alcohol consumption, history of hormone use, self-reported health, current healthcare provider), disability, comorbid conditions (diabetes mellitus, hypertension, depressed mood, history of hip fracture, falling, arthritis, cancer, COPD, coronary heart disease, CHF, stroke)
Fried et al.,2001 [4]	U.S.	7	NA	T 5317 W 3077 M 2240	All-cause NA	**FP**: Frail Prefrail Robust	Interview	age, gender, indicator for minority cohort, income, smoking status, blood pressure, fasting glucose, albumin, creatinine, carotid stenosis, history of CHF, cognitive function, major ECG abnormality, use of diuretics, problem with IADLs, self-report health measure, depression measure
Susanto et al.,2018 [43]	Australia	15	NA	W 8933	All-cause 483	**FS**: Frail Robust	National Death Index	age, BMI, educational status, ability to manage on income, physical activity
Hao et al.,2016 [79]	China	4	93.7	T 767 W 520 M 247	All-cause 395	**FI:** Severely frail (≥ 0.45) Frail (0.22–0.45) Non-frail (< 0.22) **FI:**0.01	Local government records, relatives or neighbors	age, sex, education
Theou et al.,2012 [80]	Canada	5	84.6	T 2305 W 1431 M 874	All-cause 1003	**FI:**0.1	NA	age, sex
Jacobs et al.,2011 [81]	Israel	5	85	T 840 W 440 M 400	All-cause 194	**FP**: Frail Prefrail Non-frail	Death certificate	MMSE, sex, educational status, ischemic heart disease, diabetes, hypertension, smoking status, self-rated health, ADL
Lucicesare et al.,2010 [82]	Italy	4	74.7	T 1016 W 453 M 563	All-cause 147	**FI:** Frail ≥0.25 Non-frail<0.25	Official death records	age, sex
Searle et al.,2008 [83]	U.S.	9	NA	T 754 W 487 M 267	All-cause NA	**FI:**0.01	Death certificate	age, sex
Srinonprasert et al.,2018 [84]	Thailand	7	69.2	T 8195 W 4163 M 4032	All-cause 1284	**FI:** Frail>0.25 Non-frail ≤0.25	Death Database	NA

*ADL* Activities of Daily Living, *BMI* Body Mass Index, *CHF* Congestive Heart Failure, *COPD* Chronic Obstructive Lung Disease, *COVID-19* Coronavirus Disease 2019, *CRP* C-reactive Protein, *CVD* Cardiovascular Disease, *ECG* Electrocardiograph, *FI* Frailty Index, *FP* Frailty Phenotype, *FS* FRAIL scale, *HbA1c* Glycated Hemoglobin, *HDL* High Density Lipoprotein, *IADL* Instrumental Activity of Daily Living, *LDL* Low Density Lipoprotein, *M* Men, *MMSE* Mini-Mental State Examination, *T* Total, *TDI* Townsend Deprivation Index, *U.K.* United Kingdom, *U.S.* United States, *W* Women

^a^Seven low- and middle-income countries (LMICs) including Cuba, Dominican Republic, Venezuela, Mexico, Peru, India, and China

With respect to the assessment of frailty, a total of 24 studies adopted the FP. In terms of the five established criteria, individuals meeting three or more items were regarded as frail, those meeting one or two items as prefrail, and those with no items as not frail or as robust. The FI was used in 30 studies, which usually divided the participants into two groups (frail and non-frail), three groups (frail, prefrail, and robust), and four categories (mostly frail, moderately frail, prefrail, and robust) based upon the different total numbers of baseline deficits and the different cut-off points. Moreover, 17 of these studies provided the HRs per 0.01 or per 0.1 increase in the FI and per increase in one deficit, respectively. Seven studies reported the frailty status by the FS, in which the categories of robust, prefrail, and frail were defined as individuals who had 0, 1 or 2, and 3 to 5 items, respectively.

Regarding the methodological quality, all the included studies were generally of high quality according to the NOS scale. As shown in Supplementary Table 3, all the identified studies scored from 6 to 9 points.

### Frailty and all-cause mortality

**Fig. 2:**
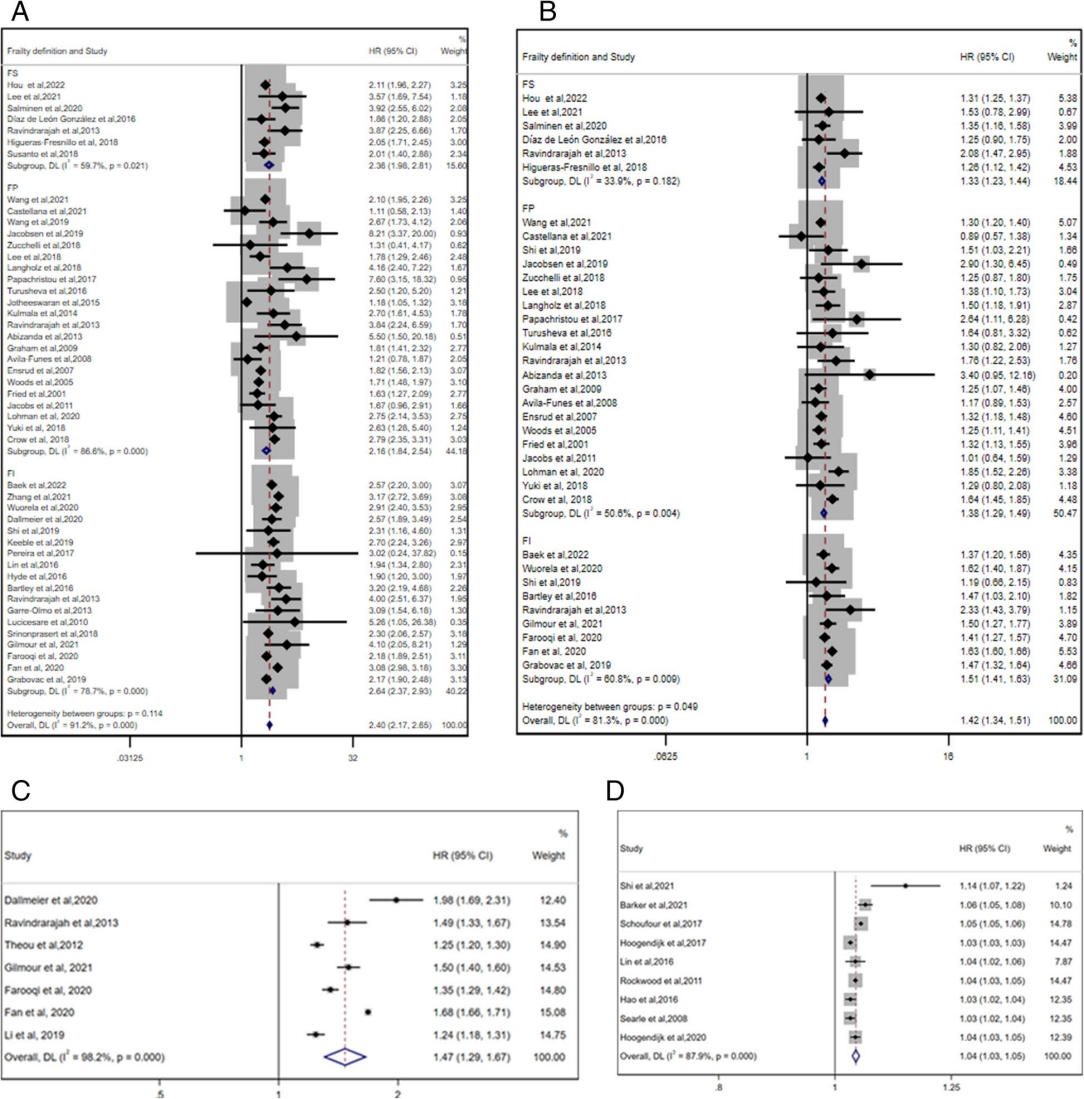
Forest plots of the all-cause mortality risk according to the frailty status. **A** The pooled HR and 95% CI of the all-cause mortality in the frail group compared with the robust group; **B** The pooled HR and 95% CI of the all-cause mortality in the prefrail group compared with the robust group; **C** The pooled HR of the all-cause mortality risk per 0.1 increase in the frailty index score; **D** The pooled HR of the all-cause mortality risk per 0.01 increase in the frailty index score. CI = confidence interval, HR = hazard ratio

Fifty-four studies [4, 26–28, 32–37, 39–49, 51–76, 78–85] were included in the meta-analysis of the association between frailty status and all-cause mortality, and the summary HRs were calculated using a random-effects model. As depicted in Fig. 2, compared with the robust group, the frail group had a significantly higher risk of all-cause mortality (pooled HR = 2.40, 95% CI 2.17–2.65; I^2^ = 91.2%, P_heterogeneity_ < .001; 48 studies; Fig. 2A). Similarly, the prefrail group also displayed a higher all-cause death risk than the robust group (pooled HR = 1.42, 95% CI 1.34–1.51; I^2^ = 81.3%, P_heterogeneity_ < .001; 36 studies; Fig. 2B). In addition, based on seven studies [27, 33–35, 37, 40, 80] that reported HRs of the all-cause mortality risk per 0.1 increase in the FI (pooled HR = 1.47, 95% CI 1.29–1.67; I^2^ = 98.2%, P_heterogeneity_ < .001; 7 studies; Fig. 2C) and nine studies [46, 54, 55, 65, 67, 69, 76, 79, 83] that reported HRs of the all-cause mortality risk per 0.01 increase in the FI (pooled HR = 1.04, 95% CI 1.03–1.05; I^2^ = 87.9%, P_heterogeneity_ < .001; 9 studies; Fig. 2D), we also confirmed that frailty was a significant predictor of all-cause mortality. In addition, one study [32] demonstrated an increased risk of all-cause death per increase in one deficit (HR = 1.04, 95% CI 1.01–1.07 for men, HR = 1.08, 95% CI 1.06–1.11 for women). These studies consistently suggested that frailty status, as defined by the FI using various ways, was linked to an increased all-cause death risk.

Considering that there was a high degree of heterogeneity across the studies, we performed further sensitivity analyses. A fixed effects model and the removal of any single study had little effect on the overall pooling risk estimate. Of note, although a similar outcome was found in subgroup analyses categorized by different frailty assessment tools (FP, FI, and FS), we found markedly decreased heterogeneity when the included studies were restricted to those using the FS to assess frailty (Fig. 2).

Marginal evidence of publication bias was found for the association between frailty status and all-cause mortality by Begg’s test and Egger’s test (Begg’s test *P* = 0.004–0.463 and Egger’s test *P* = 0.030–0.918).

### Frailty and CVD mortality

**Fig. 3:**
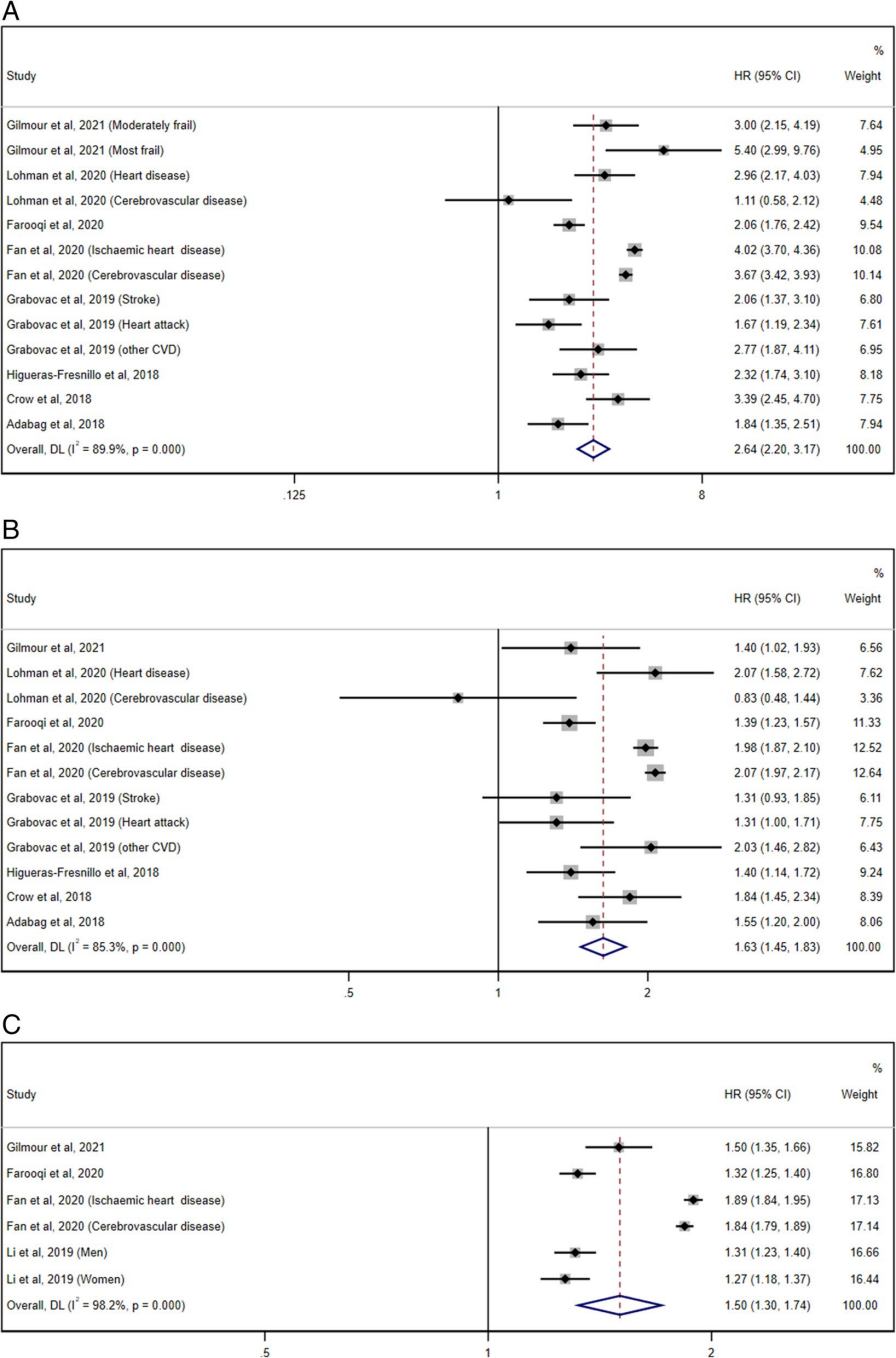
Forest plots of the CVD mortality risk according to the frailty status. **A** The pooled HR and 95% CI of the CVD mortality in the frail group compared with the robust group; **B** The pooled HR and 95% CI of the CVD mortality in the prefrail group compared with the robust group; **C** The pooled HR of the CVD mortality risk per 0.1 increase in the frailty index score. CI = confidence interval, CVD = cardiovascular disease, HR = hazard ratio

Nine studies [26–28, 34, 35, 37, 38, 48, 49] were included in the random effects meta-analysis of the effect of frailty status on the CVD mortality risk. As shown in Fig. 3, both the frail group (pooled HR = 2.64, 95% CI 2.20–3.17; I^2^ = 89.8%, P_heterogeneity_ < .001; 8 studies; Fig. 3A) and the prefrail group (pooled HR = 1.63, 95% CI 1.45–1.83; I^2^ = 85.3%, P_heterogeneity_ < .001; 8 studies; Fig. 3B) suggested an obviously increased risk for CVD death compared to the robust group with substantial heterogeneity. As expected, a similar result could be found by combining the HRs of the CVD mortality risk for each 0.1 increase in the FI (pooled HR = 1.50, 95% CI 1.30–1.74; I^2^ = 98.2%, P_heterogeneity_ < .001; 4 studies; Fig. 3C). In addition, one study [46] provided the HR of the CVD mortality risk for each 0.01 increase in the FI (HR = 1.05, 95% CI 1.03–1.06), and another study [50] reported that there was an increased CVD death risk with each increase in one deficit (HR = 1.03, 95% CI 0.99–1.08 for men, HR = 1.13, 95% CI 1.09–1.17 for women). The relationship between the frailty status as defined by the FI and the CVD mortality risk appeared to be sex-specific.

In reference to the sensitivity analysis, none of the three abovementioned methods altered the initial results. Moreover, marginal evidence of publication bias was detected for the association between frailty status and CVD mortality (Begg’s test *P* = 0.37–1.00 and Egger’s test *P* = 0.009–0.026).

### Frailty and cancer and respiratory illness mortality

**Fig. 4:**
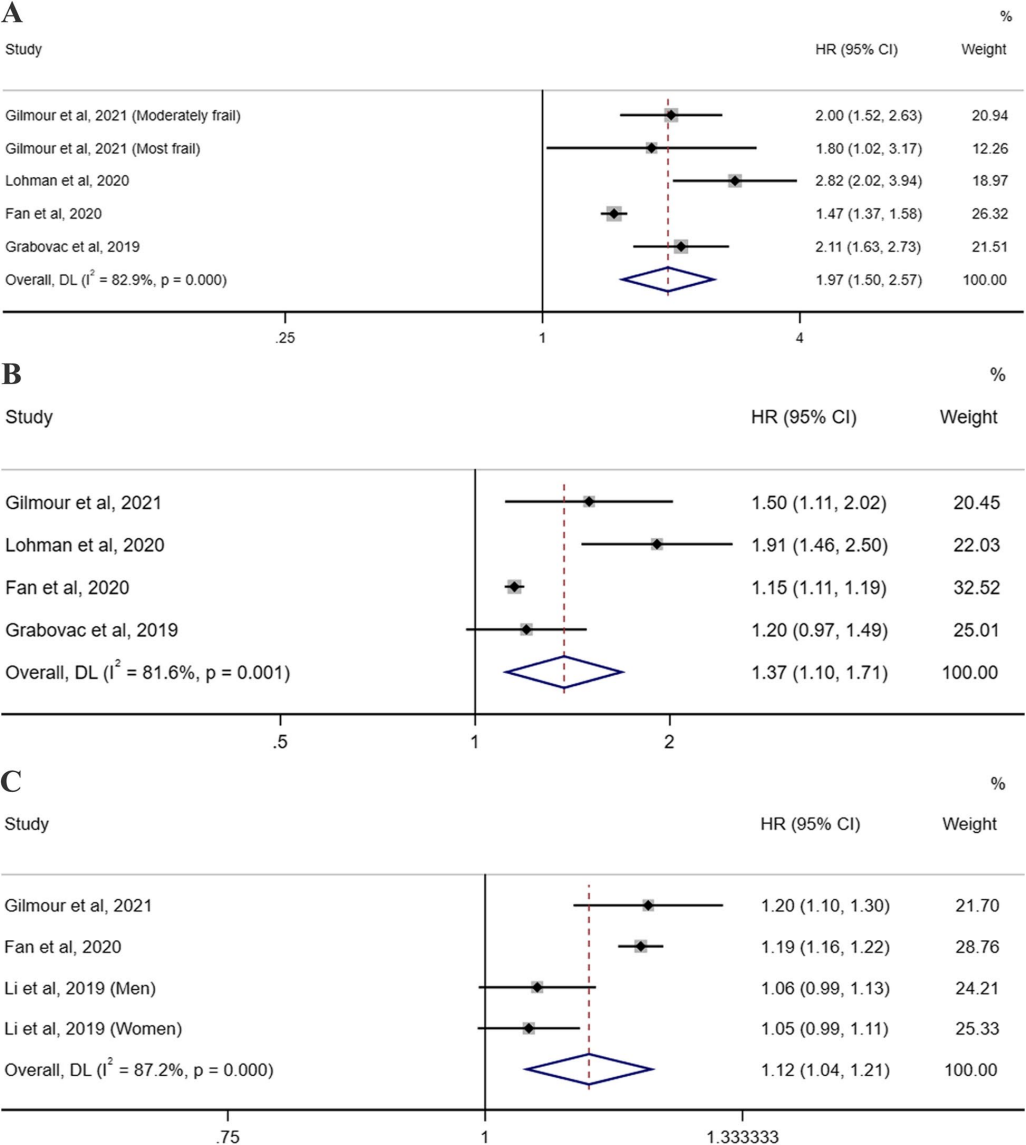
Forest plots of the cancer mortality risk according to the frailty status. **A** The pooled HR and 95% CI of the cancer mortality in the frail group compared with the robust group; **B** The pooled HR and 95% CI of the cancer mortality in the prefrail group compared with the robust group; **C** The pooled HR of the cancer mortality risk per 0.1 increase in the frailty index score. CI = confidence interval, HR = hazard ratio

**Fig. 5:**
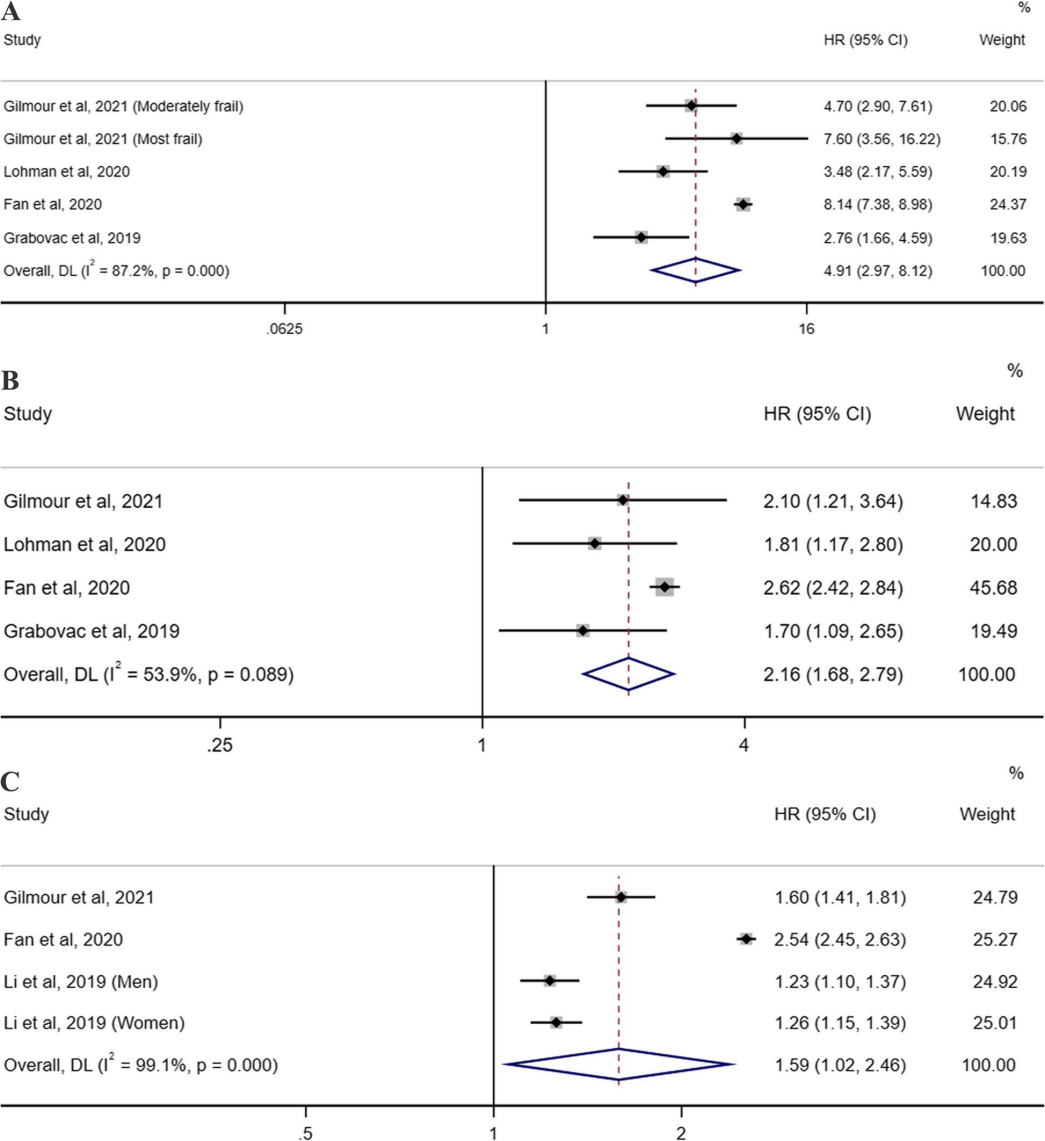
Forest plots of the respiratory illness mortality risk according to the frailty status. **A** The pooled HR and 95% CI of the respiratory illness mortality in the frail group compared with the robust group; **B** The pooled HR and 95% CI of the respiratory illness mortality in the prefrail group compared with the robust group; **C** The pooled HR of the respiratory illness mortality risk per 0.1 increase in the frailty index score. CI = confidence interval, HR = hazard ratio

Five individual studies [26–28, 34, 35] were eligible for the evaluation of the association between frailty status and cancer and respiratory illness mortality. The random-effects meta-analysis revealed that frailty could statistically increase the risk of death from cancer (pooled HR = 1.97, 95% CI 1.50–2.57; I^2^ = 82.9%, P_heterogeneity_ < .001; 4 studies; Fig. 4A) and respiratory illness (pooled HR =4.91, 95% CI 2.97–8.12; I^2^ = 87.2%, P_heterogeneity_ < .001; 4 studies; Fig. 5A). Similarly, compared with the robust group, the individuals in the prefrail group had a 1.37-fold higher risk of death from cancer (95% CI 1.10–1.71; I^2^ = 81.6%, P_heterogeneity_ = 0.001; 4 studies; Fig. 4B) and a 2.16-fold higher risk of death from respiratory illness (95% CI 1.68–2.79; I^2^ = 53.9%, P_heterogeneity_ = 0.089; 4 studies; Fig. 5B). In addition, with the three studies [27, 34, 35] that reported the HRs of the cancer and respiratory illness mortality risk per 0.1 increase in the FI, a pooled HR of 1.12 (95% CI = 1.04–1.21; I^2^ = 87.2%, P_heterogeneity_ < .001; 3 studies; Fig. 4C) for cancer death and 1.59 (95% CI = 1.02–2.46; I^2^ = 99.1%, P_heterogeneity_ < .001; 3 studies; Fig. 5C) for respiratory illness death was obtained.

## Discussion

Previous evidence [19, 20, 22] has shown that there is a significant association between frailty and all-cause mortality. The present study revealed positive correlations between frailty, prefrailty, and all-cause mortality and further demonstrated that frailty was a strong predictor of cause-specific mortality from CVD, cancer, and respiratory illness. To the best of our knowledge, this is the first systematic review and meta-analysis to explore the influence that frailty exerts on cause-specific mortality among adults living in communities. Specifically, we found an almost 2-fold increased risk in the frail group and a 1.5-fold increased risk in the prefrail group for all-cause mortality, CVD mortality, and cancer mortality, respectively. Of note, the risk of respiratory illness mortality was approximately doubled in both the frail and prefrail groups, with 4.91- and 2.16-fold higher risks compared to the robust group. In addition, the all-cause and cause-specific mortality risk per 0.1 and per 0.01 increase in the FI showed consistently significant results, which indicated that the risk of death increased with the increase in the frailty status (i.e., from prefrailty worsening to frailty).

We did not perform a meta-analysis on the association between frailty status and mortality from dementia, infection, and COVID-19 due to the limited number of studies, but the research outcomes were still noteworthy. The study by Lohman et al. [26] suggested that frailty was associated with a 2.87 (95% CI = 1.47–5.59) times greater hazard of death from dementia, while prefrailty was not a predictor of dementia mortality. In addition, Jiang et al. [50] found that frailty, as defined by the FI, was not linked to dementia mortality in either sex. Two identified studies [27, 28] independently confirmed that frailty and prefrailty were associated with a higher risk of mortality due to infection. Nevertheless, the statistically significant association between prefrailty and the infection mortality disappeared after adjusting for all the confounding factors, as seen in the further analyses in one study [28]. During the global pandemic of COVID-19, a plethora of studies [15, 86, 87] have reported that an increased COVID-19 mortality risk has been associated with frailty. However, most of these studies focused on patients who were diagnosed with COVID-19 until a recent study by Mak et al., [25] which found that frailty was related to a higher COVID-19 mortality risk in a community population. A dramatically growing number of confirmed cases has raised public attention to determine the effect of frailty on COVID-19 mortality in the general population. The number of studies that evaluated the associations of frailty with dementia, infection, and COVID-19 mortality has been too small to yield reliable results. Thus, more congeneric studies are warranted.

Although substantial heterogeneity existed in our meta-analysis, we only found that the different frailty assessment tools might be the underlying effect factor in the sensitivity analysis. We included studies that defined frailty using one of three widely used tools: the FP, FI, and FS. In fact, when we restricted the includes studies to those only using the FS, the heterogeneity notably declined. There is no consensus regarding the gold standard to assess frailty to date. In recent decades, a vast variety of frailty assessment tools, [4, 77, 88–90] such as the FP, FI, FS, Groningen Frailty Indicator, Tilburg Frailty Indicator, Clinical Frailty Scale, etc., have been proposed and well validated. However, some comparative studies [7, 91] have also found substantive differences between these tools in their validity, feasibility, and ability to predict mortality. A 2017 umbrella review [21] examined five systematic reviews to compare the reliability, validity, accuracy, and predictive ability of 34 frailty screening tools in older adults, and found that the FI had good predictive ability and mostly acceptable validity and diagnostic accuracy. Notably, significant heterogeneity was found in the subgroups using the FI as the frailty assessment method (as shown in Fig. 2), which is consistent with a previous systematic review of the all-cause mortality risk according to the FI [16]. First, the FI was constructed based on the different numbers and types of deficits. In addition, the included studies defined frailty with different cut-off points for the FI. Therefore, to reduce the heterogeneity across studies, we need more studies with uniform frailty assessment tools.

Previous research has explored sex and age effects on the association between frailty and mortality risk, but the results are still in dispute. Some studies [17, 50, 92] have found a sex-specific impact of frailty on all-cause and cause-specific mortality. However, a recent meta-analysis found that there was no sex difference in the association of frailty with mortality [93]. In addition, shorter follow-up periods and younger age were found to be potentially associated with a higher mortality risk [16, 50]. However, neither sex nor age or follow-up duration showed any effect on the relationship between frailty and all-cause or cause-specific mortality in the current study. Hence, more large-scale studies are required to identify whether sex, the age threshold, or the follow-up duration can modify the frailty-mortality association.

Several limitations should be considered in our meta-analysis. First, significant heterogeneity was observed in the statistical analysis, which caused concerns about the reliability of the pooled results. However, through sensitivity analyses, we found that the sources of heterogeneity could be partially explained by the different assessment tools adopted to measure frailty. Although the different measuring methods and cut-off points across studies could possibly lead to a misclassification of frailty, the subgroup analysis based on the different frailty assessment tools showed consistent results. Additionally, irrespective of which tools were used to define frailty, both frailty and prefrailty were significantly associated with a higher mortality risk in previous studies [16–18]. Moreover, methodological heterogeneity was inevitable in all the meta-analyses, especially the meta-analyses based on observational studies. Second, frailty is a dynamic process that usually progresses to greater frailty (i.e., “worsening”) with ageing but could be reversible by effective interventions [94, 95]. However, because only the baseline frailty status was evaluated in the included studies, we could not overcome the confounding effects from the progression of frailty during the follow-up duration. In addition, even though we extracted the most fully adjusted risk estimates, residual confounding still existed. Third, most included studies ascertained the causes of death from a death registry or a national death database according to the International Classification of Diseases (ICD) codes; thus, inaccurate information under some circumstances might cause a misclassification bias. Fourth, we restricted our search to studies published in English, and this is a possible source of bias. Finally, the number of studies of cause-specific mortality was limited, especially for studies that included cancer and respiratory illness mortality. This hampered further analysis, given that these analysis results were potentially unreliable under the condition that the number of identified studies was less than 10, especially in the sensitivity, subgroup, or meta-regression analyses. Additionally, we only performed a systematic review but not a meta-analysis since there were fewer studies on the associations between frailty and dementia, infection, and COVID-19 mortality.

## Conclusion

In conclusion, frailty was not only significantly associated with an increased risk of all-cause mortality but was also a strong predictor of cause-specific mortality from CVD, cancer, and respiratory illness in community-dwelling adults. These findings highlight the importance of frailty interventions in reducing the risk of death in the general population and indicate which population will benefit the most from efficient interventions. Early diagnosis of frailty can help identify high-risk older adults, helping to minimize the risk of prefrail status developing into frail status and even reverse frailty status. In addition, the implementation of therapeutic measures such as physical activity, nutrition support, comorbidities and polypharmacy management could reduce disability, institutionalization, hospitalization, the need for long-term care, medical and social costs, and death. Furthermore, knowing the increased risk stratified by cause of death allows us to make further targeted interventions regarding the natural development of frailty status as well as aid in designing disease-specific interventions to reduce mortality. Nonetheless, these results should be interpreted with caution due to the limited number of studies included in our meta-analysis; thus, more studies are warranted in the future to explore the association of frailty with cause-specific mortality.

## Supplementary Information

**Additional file 1: Supplementary Table 1.** Search strategy.

**Additional file 2: Supplementary Table2.** The list of studies excluded after full-text review.

**Additional file 3: Supplementary Table 3.** The results of quality assessment for the included studies.

## Data Availability

All data generated or analysed during this study are available from the included studies in this article.

## Abbreviations

CIs: Confidence intervals
COVID-19: Coronavirus disease 2019
CVD: Cardiovascular disease
FI: Frailty index
FP: Frailty phenotype
FS: FRAIL scale
HR: Hazard ratio
ICD: International Classification of Diseases
NOS: Newcastle-Ottawa Quality Assessment Scale
OR: Odds ratio
