# Supplementary material for: Frailty and risks of all-cause and cause-specific death in community-dwelling adults: a systematic review and meta-analysis
Source: BMC Geriatr. 2022 Sep 2;22:725. doi: 10.1186/s12877-022-03404-w (PMC9437382; doi:10.1186/s12877-022-03404-w)
Supplement: Supplementary file 1 — Additional file 1: Supplementary Table 1. Search strategy. [file 12877_2022_3404_MOESM1_ESM.doc]

**Supplementary Table 1** Search strategy

| **Search strategy for Embase** |
| --- |
| #1 'frailty':ab,ti OR 'frail':ab,ti 43,840  #2 'mortality':ab,ti OR 'death':ab,ti 2,233,375  #3 'english':la AND [humans]/lim 21,255,477  #4 #1 AND #2 AND #3 12,633  #5 'review':it OR 'letter':it OR 'editorial':it OR 'erratum':it 5,129,605  #6 #4 NOT #5 11,349 |
| **Search strategy for Pubmed** |
| #1 frailty[Title/Abstract] OR frail[Title/Abstract] 30,202  #2 mortality[Title/Abstract] OR death[Title/Abstract] 1,564,280  #3 English[Language] AND Humans[Filter] 16,899,982  #4 #1 AND #2 AND #3 6,533  #5 Review[Publication Type] OR Letter[Publication Type] OR Editorial[Publication Type] OR Published erratum[Publication Type] 4,870,360  #6 #4 NOT #5 5,348 |
