# Supplementary material for: Frailty and risks of all-cause and cause-specific death in community-dwelling adults: a systematic review and meta-analysis
Source: BMC Geriatr. 2022 Sep 2;22:725. doi: 10.1186/s12877-022-03404-w (PMC9437382; doi:10.1186/s12877-022-03404-w)
Supplement: Supplementary file 2 — Additional file 2: Supplementary Table2. The list of studies excluded after full-text review. [file 12877_2022_3404_MOESM2_ESM.docx]

**Supplementary Table 2 The list of studies excluded after full-text review**

**Studies excluded after full-text review: N= 79**

Cross-sectional analysis or conference abstract: N= 12

[1],[2],[3],[4],[5],[6],[7],[8],[9],[10],[11],[12]

Same cohort studies: N= 23

[13],[14],[15],[16],[17],[18],[19],[20],[21],[22],[23],[24],[25],[26],[27],[28],[29],[30],[31],[32],[33],[34],[35]

Not community dwelling participants: N= 7

[36],[37],[38],[39],[40],[41],[42]

The exposure is change of frailty or combined impact with other factors: N= 10

[43],[44],[45],[46],[47],[48],[49],[50],[51],[52]

Non-standard definition or classification of frailty: N= 10

[53],[54],[55],[56],[57],[58],[59],[60],[61],[62]

Required risk estimates and 95% CIs were not available: N= 17

[63],[64],[65],[66],[67],[68],[69],[70],[71],[72],[73],[74],[75],[76],[77],[78],[79]

1. Barker FJ, Davies JI, Gomez-Olive FX, Kahn K, Matthews FE, Payne CF, et al. A deficit accumulation frailty index predicts mortality in older South Africans: Findings from the Haalsi study. Age and Ageing. 2020;49.

2. Khadka J, Visvanathan R, Theou O, Wesselingh S, Inacio MC. Development and validation of a national frailty index. Journal of the American Geriatrics Society. 2019;67:S147.

3. Akın S, Özer FF, Ertürk G, Göçer S, Mazıcıoğlu MM, Şafak ED, et al. Which frailty scale predicts 4-year mortality in community-dwelling turkish elderly better: The FRAIL scale or the fried frailty index? Erciyes Medical Journal. 2019;41(1):56-61.

4. Zasadzka E, Roszak M, Trzmiel T, Pawlaczyk M. Stages of frailty syndrome and mortality in older men and women 80+. European Geriatric Medicine. 2018;9:S94.

5. Keeble E, Neuburger J, Arora S, Jagger C, Duncan R, Robinson AL, et al. Frailty and mortality, hospital use and costs over 7 years in the Newcastle 85+ cohort. European Geriatric Medicine. 2018;9:S103.

6. Hoogendijk EO, Jeuring HW, Huisman M, Deeg DJH. Trends in frailty and its association with mortality across a period of 21 years: Results from the longitudinal aging study Amsterdam. European Geriatric Medicine. 2018;9:S89.

7. Langholz PL, Strand BH, Cook S, Hopstock LA. The frailty phenotype as a predictor of all-cause mortality in community-living individuals aged 70 years and older: The Tromso Study 2001-2016. Norsk Epidemiologi. 2017;27:78.

8. Crow RS, Lohman MC, Bruce ML, Mackenzie TA, Batsis JA. Mortality risk along the frailty spectrum: Data from NHANES 1999-2004. Journal of the American Geriatrics Society. 2017;65:S77.

9. Kim SY, Won CW, Lee HY. The effect of frailty on 3-year mortality in Korean community-dwelling elderly. European Geriatric Medicine. 2015;6:S92.

10. Ravindrarajah R, Lee DM, Pye SR, Pendleton N, Finn JD, O'Connell MDL, et al. Frailty is linked with an increased mortality in European men. Age and Ageing. 2012;41:ii54.

11. Zhang Q, Guo H, Gu H, Zhao X. Gender-associated factors for frailty and their impact on hospitalization and mortality among community- dwelling older adults: A cross-sectional population-based study. PeerJ. 2018;2018(2).

12. Adabag S, Vo TN, Taylor BC, Langsetmo L, Schousboe JT, Kats A, et al. Frailty as a risk factor for cardiovascular versus non-cardiovascular mortality in elderly men: Results from the outcomes of sleep disorders in older men (MrOS sleep) study. Journal of the American College of Cardiology. 2017;69(11):1785.

13. Zhang J, Cao X, Chen C, He L, Ren Z, Xiao J, et al. Predictive Utility of Mortality by Aging Measures at Different Hierarchical Levels and the Response to Modifiable Life Style Factors: Implications for Geroprotective Programs. Frontiers in Medicine. 2022;9.

14. Petermann-Rocha F, Pell JP, Celis-Morales C, Ho FK. Frailty, sarcopenia, cachexia and malnutrition as comorbid conditions and their associations with mortality: a prospective study from UK Biobank. Journal of public health (Oxford, England). 2021.

15. Petermann-Rocha F, Gray SR, Pell JP, Ho FK, Celis-Morales C. The joint association of sarcopenia and frailty with incidence and mortality health outcomes: A prospective study. Clinical Nutrition. 2021;40(4):2427-34.

16. Midão L, Brochado P, Almada M, Duarte M, Paúl C, Costa E. Frailty status and polypharmacy predict all-cause mortality in community dwelling older adults in Europe. International Journal of Environmental Research and Public Health. 2021;18(7).

17. Conde-Sala JL, Garre-Olmo J, Calvó-Perxas L, Turró-Garriga O, Vilalta-Franch J, López-Pousa S. CAUSES, mortality rates and risk factors of death in community-dwelling Europeans aged 50 years and over: Results from the Survey of Health, Ageing and Retirement in Europe 2013-2015. Arch Gerontol Geriatr. 2020;89:104035.

18. Hanlon P, Nicholl BI, Jani BD, Lee D, McQueenie R, Mair FS. Frailty and pre-frailty in middle-aged and older adults and its association with multimorbidity and mortality: a prospective analysis of 493 737 UK Biobank participants. The Lancet Public Health. 2018;3(7):e323-e32.

19. Wang C, Ji X, Wu X, Tang Z, Zhang X, Guan S, et al. Frailty in relation to the risk of Alzheimer’s disease, dementia, and death in older Chinese adults: A seven-year prospective study. Journal of Nutrition, Health and Aging. 2017;21(6):648-54.

20. Payne CF, Wade A, Kabudula CW, Davies JI, Chang AY, Gomez-Olive FX, et al. Prevalence and correlates of frailty in an older rural African population: findings from the HAALSI cohort study. BMC geriatrics. 2017;17(1):293.

21. Kane AE, Gregson E, Theou O, Rockwood K, Howlett SE. The association between frailty, the metabolic syndrome, and mortality over the lifespan. GeroScience. 2017;39(2):221-9.

22. Gu D, Feng Q. Frailty still matters to health and survival in centenarians: the case of China. BMC geriatrics. 2015;15:159.

23. Romero-Ortuno R, Soraghan C. A Frailty Instrument for primary care for those aged 75 years or more: Findings from the Survey of Health, Ageing and Retirement in Europe, a longitudinal population-based cohort study (SHARE-FI75+). BMJ Open. 2014;4(12).

24. Llibre Jde J, López AM, Valhuerdi A, Guerra M, Llibre-Guerra JJ, Sánchez YY, et al. Frailty, dependency and mortality predictors in a cohort of Cuban older adults, 2003-2011. MEDICC Rev. 2014;16(1):24-30.

25. Lahousse L, Maes B, Ziere G, Loth DW, Verlinden VJA, Zillikens MC, et al. Adverse outcomes of frailty in the elderly: The Rotterdam Study. European Journal of Epidemiology. 2014;29(6):419-27.

26. Yu P, Song X, Shi J, Mitnitski A, Tang Z, Fang X, et al. Frailty and survival of older Chinese adults in urban and rural areas: Results from the Beijing Longitudinal Study of Aging. Archives of Gerontology and Geriatrics. 2012;54(1):3-8.

27. Fang X, Shi J, Song X, Mitnitski A, Tang Z, Wang C, et al. Frailty in relation to the risk of falls, fractures, and mortality in older Chinese adults: results from the Beijing Longitudinal Study of Aging. J Nutr Health Aging. 2012;16(10):903-7.

28. Song X, Mitnitski A, Rockwood K. Prevalence and 10-Year outcomes of frailty in older adults in relation to deficit accumulation. Journal of the American Geriatrics Society. 2010;58(4):681-7.

29. Romero-Ortuno R, Walsh CD, Lawlor BA, Kenny RA. A frailty instrument for primary care: findings from the Survey of Health, Ageing and Retirement in Europe (SHARE). BMC geriatrics. 2010;10:57.

30. Gu D, Dupre ME, Sautter J, Zhu H, Liu Y, Yi Z. Frailty and mortality among chinese at advanced ages. Journals of Gerontology - Series B Psychological Sciences and Social Sciences. 2009;64(2):279-89.

31. García-González JJ, García-Peña C, Franco-Marina F, Gutiérrez-Robledo LM. A frailty index to predict the mortality risk in a population of senior Mexican adults. BMC Geriatr. 2009;9:47.

32. Ensrud KE, Ewing SK, Cawthon PM, Fink HA, Taylor BC, Cauley JA, et al. A comparison of frailty indexes for the prediction of falls, disability, fractures, and mortality in older men. Journal of the American Geriatrics Society. 2009;57(3):492-8.

33. Berges IM, Graham JE, Ostir GV, Markides KS, Ottenbacher KJ. Sex differences in mortality among older frail Mexican Americans. Journal of Women's Health. 2009;18(10):1647-51.

34. Cawthon PM, Marshall LM, Michael Y, Dam TT, Ensrud KE, Barrett-Connor E, et al. Frailty in older men: Prevalence, progression, and relationship with mortality. Journal of the American Geriatrics Society. 2007;55(8):1216-23.

35. Jones D, Song X, Mitnitski A, Rockwood K. Evaluation of a frailty index based on a comprehensive geriatric assessment in a population based study of elderly Canadians. Aging Clin Exp Res. 2005;17(6):465-71.

36. Varan HD, Kizilarslanoglu MC, Dogrul RT, Arik G, Esme M, Kara O, et al. Comparative Evaluation of Predictive Ability of Comprehensive Geriatric Assessment Components Including Frailty on Long-Term Mortality. Experimental aging research. 2021;47(3):220-31.

37. Li G, Ioannidis G, Pickard L, Kennedy C, Papaioannou A, Thabane L, et al. Frailty index of deficit accumulation and falls: Data from the Global Longitudinal Study of Osteoporosis in Women (GLOW) Hamilton cohort. BMC Musculoskeletal Disorders. 2014;15(1).

38. Hogan DB, Freiheit EA, Strain LA, Patten SB, Schmaltz HN, Rolfson D, et al. Comparing frailty measures in their ability to predict adverse outcome among older residents of assisted living. BMC geriatrics. 2012;12:56.

39. Sirola J, Pitkala KH, Tilvis RS, Miettinen TA, Strandberg TE. Definition of frailty in older men according to questionnaire data (RAND-36/SF-36): The Helsinki Businessmen study. Journal of Nutrition, Health and Aging. 2011;15(9):783-7.

40. Bandeen-Roche K, Xue QL, Ferrucci L, Walston J, Guralnik JM, Chaves P, et al. Phenotype of frailty: Characterization in the Women's Health and Aging Studies. Journals of Gerontology - Series A Biological Sciences and Medical Sciences. 2006;61(3):262-6.

41. Sanchis J, Bonanad C, Ruiz V, Fernández J, García-Blas S, Mainar L, et al. Frailty and other geriatric conditions for risk stratification of older patients with acute coronary syndrome. American Heart Journal. 2014.

42. Wienke A, Holm NV, Skytthe A, Yashin AI. The heritability of mortality due to heart diseases: A correlated frailty model applied to Danish twins. Twin Research. 2001;4(4):266-74.

43. Xue QL, Bandeen-Roche K, Tian J, Kasper JD, Fried LP. Progression of Physical Frailty and the Risk of All-Cause Mortality: Is There a Point of No Return? Journal of the American Geriatrics Society. 2021;69(4):908-15.

44. Thompson MQ, Yu S, Tucker GR, Adams RJ, Cesari M, Theou O, et al. Frailty and sarcopenia in combination are more predictive of mortality than either condition alone. Maturitas. 2021;144:102-7.

45. Strandberg TE, Lindström L, Jyväkorpi S, Urtamo A, Pitkälä KH, Kivimäki M. Phenotypic frailty and multimorbidity are independent 18-year mortality risk indicators in older men: The Helsinki Businessmen Study (HBS). European Geriatric Medicine. 2021.

46. Ruiz-Grao MC, Sánchez-Jurado PM, Molina-Alarcón M, Hernández-Martínez A, Avendaño Céspedes A, Abizanda P. Frailty, depression risk, and 10-year mortality in older adults: The FRADEA study. International Psychogeriatrics. 2020.

47. Hoogendijk EO, Smit AP, van Dam C, Schuster NA, de Breij S, Holwerda TJ, et al. Frailty Combined with Loneliness or Social Isolation: An Elevated Risk for Mortality in Later Life. Journal of the American Geriatrics Society. 2020;68(11):2587-93.

48. Aliberti MJR, Cenzer IS, Smith AK, Lee SJ, Yaffe K, Covinsky KE. Assessing Risk for Adverse Outcomes in Older Adults: The Need to Include Both Physical Frailty and Cognition. Journal of the American Geriatrics Society. 2019;67(3):477-83.

49. St John PD, Tyas SL, Griffith LE, Menec V. The cumulative effect of frailty and cognition on mortality - Results of a prospective cohort study. International Psychogeriatrics. 2017;29(4):535-42.

50. Taniguchi Y, Kitamura A, Abe T, Kojima G, Shinozaki T, Seino S, et al. Associations of aging trajectories for an index of frailty score with mortality and medical and long-term care costs among older Japanese undergoing health checkups. Geriatrics and Gerontology International. 2020;20(11):1072-8.

51. Liu ZY, Wei YZ, Wei LQ, Jiang XY, Wang XF, Shi Y, et al. Frailty transitions and types of death in Chinese older adults: A population-based cohort study. Clinical Interventions in Aging. 2018;13:947-56.

52. Morley JE, Malmstrom TK, Miller DK. A simple frailty questionnaire (Frail) predicts outcomes in middle aged African Americans. Journal of Nutrition, Health and Aging. 2012:1-8.

53. Diniz MA, Rodrigues RAP, Fhon JRS, Haas VJ, Fabrício-Wehbe SCC, Giacomini SBL, et al. Frailty and its relationship to mortality among older adults from a Brazilian community: A cohort study. Journal of Clinical Gerontology and Geriatrics. 2018;9(1):27-33.

54. Buchman AS, Leurgans SE, Boyle PA, Schneider JA, Arnold SE, Bennett DA. Combinations of motor measures more strongly predict adverse health outcomes in old age: The rush memory and aging project, a community-based cohort study. BMC Medicine. 2011;9.

55. Puts MT, Lips P, Deeg DJ. Sex differences in the risk of frailty for mortality independent of disability and chronic diseases. J Am Geriatr Soc. 2005;53(1):40-7.

56. Olivieri-Mui BL, Shi SM, McCarthy EP, Habtemariam D, Kim DH. Beyond the Health Deficit Count: Examining Deficit Patterns in a Deficit-Accumulation Frailty Index. Journal of the American Geriatrics Society. 2021;69(3):792-7.

57. Kim YP, Choe YR, Park JH, Kim S, Won CW, Hwang HS. Frailty index associated with all-cause mortality, long-term institutionalization, and hip fracture. European Geriatric Medicine. 2019;10(3):403-11.

58. Armstrong JJ, Mitnitski A, Launer LJ, White LR, Rockwood K. Frailty in the Honolulu-Asia Aging Study: deficit accumulation in a male cohort followed to 90% mortality. The journals of gerontology Series A, Biological sciences and medical sciences. 2015;70(1):125-31.

59. Saum KU, Dieffenbach AK, Müller H, Holleczek B, Hauer K, Brenner H. Frailty prevalence and 10-year survival in community-dwelling older adults: Results from the ESTHER cohort study. European Journal of Epidemiology. 2014;29(3):171-9.

60. Kamaruzzaman S, Ploubidis GB, Fletcher A, Ebrahim S. A reliable measure of frailty for a community dwelling older population. Health and Quality of Life Outcomes. 2010;8.

61. Klein BEK, Klein R, Knudtson MD, Lee KE. Frailty, morbidity and survival. Archives of Gerontology and Geriatrics. 2005;41(2):141-9.

62. Vaes B, Depoortere D, Van Pottelbergh G, Matheï C, Neto J, Degryse J. Association between traditional cardiovascular risk factors and mortality in the oldest old: untangling the role of frailty. BMC geriatrics. 2017;17(1):234.

63. Romero-Ortuno R, Hartley P, Kenny RA, O'Halloran A. 'Frail' by different measures: a comparison of 8-year mortality in the irish longitudinal study on ageing (TILDA). Age and ageing. 2021;50(SUPPL 3).

64. Hoogendijk EO, Stolz E, Oude Voshaar RC, Deeg DJH, Huisman M, Jeuring HW. Trends in Frailty and Its Association With Mortality: Results From the Longitudinal Aging Study Amsterdam, 1995-2016. American journal of epidemiology. 2021;190(7):1316-23.

65. Fustinoni S, Santos-Eggimann B, Henchoz Y. Does the frailty phenotype at the age of 66 to 71 predict death? A 14-year survival analysis of the Lc65+ study. Swiss medical weekly. 2021;151(35-36).

66. Oude Voshaar RC, Jeuring HW, Borges MK, van den Brink RHS, Marijnissen RM, Hoogendijk EO, et al. Course of frailty stratified by physical and mental multimorbidity patterns: a 5-year follow-up of 92,640 participants of the LifeLines cohort study. BMC Medicine. 2021;19(1).

67. Pereira AA, Borim FSA, Aprahamian I, Neri AL. Comparison of Two Models of Frailty for the Prediction of Mortality in Brazilian Community-Dwelling Older Adults: The FIBRA Study. Journal of Nutrition, Health and Aging. 2019;23(10):1004-10.

68. Burn R, Hubbard RE, Scrase RJ, Abey-Nesbit RK, Peel NM, Schluter PJ, et al. A frailty index derived from a standardized comprehensive geriatric assessment predicts mortality and aged residential care admission. BMC geriatrics. 2018;18(1):319.

69. Perttila NM, Pitkala KH, Kautiainen H, Tilvis R, Stranberg T. Various Diagnostic Measures of Frailty as Predictors for Falls, Weight Change, Quality of Life, and Mortality among Older Finnish Men. The Journal of frailty & aging. 2017;6(4):188-94.

70. Gray WK, Orega G, Kisoli A, Rogathi J, Paddick SM, Longdon AR, et al. Identifying Frailty and its Outcomes in Older People in Rural Tanzania. Experimental aging research. 2017;43(3):257-73.

71. Cornman JC, Glei DA, Goldman N, Weinstein M. Physiological Dysregulation, Frailty, and Risk of Mortality Among Older Adults. Research on aging. 2017;39(8):911-33.

72. Malmstrom TK, Miller DK, Morley JE. A comparison of four frailty models. Journal of the American Geriatrics Society. 2014;62(4):721-6.

73. Woo J, Leung J, Morley JE. Comparison of frailty indicators based on clinical phenotype and the multiple deficit approach in predicting mortality and physical limitation. Journal of the American Geriatrics Society. 2012;60(8):1478-86.

74. Hyde Z, Flicker L, Almeida OP, Hankey GJ, McCaul KA, Chubb SA, et al. Low free testosterone predicts frailty in older men: the health in men study. The Journal of clinical endocrinology and metabolism. 2010;95(7):3165-72.

75. Song X, MacKnight C, Latta R, Mitnitski AB, Rockwood K. Frailty and survival of rural and urban seniors: Results from the Canadian study of health and aging. Aging - Clinical and Experimental Research. 2007;19(2):145-53.

76. Mitnitski A, Song X, Skoog I, Broe GA, Cox JL, Grunfeld E, et al. Relative fitness and frailty of elderly men and women in developed countries and their relationship with mortality. Journal of the American Geriatrics Society. 2005;53(12):2184-9.

77. Sergi G, Veronese N, Fontana L, De Rui M, Bolzetta F, Zambon S, et al. Pre-frailty and risk of cardiovascular disease in elderly men and women: The Pro.V.A. Study. Journal of the American College of Cardiology. 2015;65(10):976-83.

78. Peña FG, Theou O, Wallace L, Brothers TD, Gill TM, Gahbauer EA, et al. Comparison of alternate scoring of variables on the performance of the frailty index. BMC geriatrics. 2014;14:25.

79. Kulminski AM, Ukraintseva SV, Kulminskaya IV, Arbeev KG, Land K, Yashin AI. Cumulative deficits better characterize susceptibility to death in elderly people than phenotypic frailty: Lessons from the cardiovascular health study. Journal of the American Geriatrics Society. 2008;56(5):898-903.
