# Supplementary material for: Frailty and risks of all-cause and cause-specific death in community-dwelling adults: a systematic review and meta-analysis
Source: BMC Geriatr. 2022 Sep 2;22:725. doi: 10.1186/s12877-022-03404-w (PMC9437382; doi:10.1186/s12877-022-03404-w)
Supplement: Supplementary file 3 — Additional file 3: Supplementary Table 3. The results of quality assessment for the included studies. [file 12877_2022_3404_MOESM3_ESM.doc]

| **Supplementary Table 3** The results of quality assessment for the included studies. | | | | | | | | | | | |
| --- | --- | --- | --- | --- | --- | --- | --- | --- | --- | --- | --- |
| Study | Selection | | | | Comparability | | Outcome | | | | Total Score |
|  | Representativeness of exposed cohort  ☆ | Selection of non-exposed cohort  ☆ | Exposure ascertainment  ☆ | No Death at enrollment  ☆ | Comparable  on confounders  ☆☆ | | Outcome  Assessment  ☆ | Adequate follow-up  (≥5y)  ☆ | | Loss to follow-up rate (≤20%)  ☆ |  |
| Mak et al, 2021 | ☆ | ☆ | ☆ | ☆ | ☆ | ☆ | ☆ | |  | ☆ | 8 |
| Gilmour et al, 2021 | ☆ | ☆ | ☆ | ☆ | ☆ | ☆ | ☆ | |  | ☆ | 8 |
| Lohman et al, 2020 | ☆ | ☆ | ☆ | ☆ | ☆ | ☆ | ☆ | | ☆ | ☆ | 9 |
| Hoogendijk et al, 2020 | ☆ | ☆ | ☆ | ☆ | ☆ | ☆ | ☆ | | ☆ | ☆ | 9 |
| Farooqi et al, 2020 |  | ☆ | ☆ | ☆ | ☆ | ☆ | ☆ | |  |  | 6 |
| Fan et al, 2020 | ☆ | ☆ | ☆ | ☆ | ☆ | ☆ | ☆ | | ☆ | ☆ | 9 |
| Li et al, 2019 |  | ☆ | ☆ | ☆ | ☆ | ☆ | ☆ | | ☆ | ☆ | 8 |
| Grabovac et al, 2019 | ☆ | ☆ | ☆ | ☆ | ☆ | ☆ | ☆ | | ☆ | ☆ | 9 |
| Yuki et al, 2018 | ☆ | ☆ | ☆ | ☆ | ☆ | ☆ | ☆ | | ☆ |  | 8 |
| Higueras-Fresnillo et al, 2018 | ☆ | ☆ | ☆ | ☆ | ☆ | ☆ | ☆ | | ☆ | ☆ | 9 |
| Crow et al, 2018 | ☆ | ☆ | ☆ | ☆ | ☆ | ☆ | ☆ | | ☆ | ☆ | 9 |
| Adabag et al, 2018 |  | ☆ | ☆ | ☆ | ☆ | ☆ | ☆ | | ☆ | ☆ | 8 |
| Jiang et al, 2017 |  | ☆ | ☆ | ☆ | ☆ | ☆ | ☆ | | ☆ | ☆ | 8 |
| Hou et al,2022 | ☆ | ☆ | ☆ | ☆ | ☆ | ☆ | ☆ | | ☆ | ☆ | 9 |
| Baek et al,2022 | ☆ | ☆ | ☆ | ☆ | ☆ | ☆ | ☆ | | ☆ |  | 8 |
| Zhang et al,2021 | ☆ | ☆ |  | ☆ | ☆ | ☆ | ☆ | | ☆ |  | 7 |
| Wang et al,2021 | ☆ | ☆ | ☆ | ☆ | ☆ | ☆ |  | |  | ☆ | 7 |
| Shi et al,2021 | ☆ | ☆ | ☆ | ☆ | ☆ | ☆ | ☆ | | ☆ | ☆ | 9 |
| Barker et al,2021 | ☆ | ☆ | ☆ | ☆ | ☆ |  |  | |  | ☆ | 6 |
| Lee et al,2021 | ☆ | ☆ | ☆ | ☆ | ☆ | ☆ |  | |  | ☆ | 7 |
| Castellana et al,2021 | ☆ | ☆ | ☆ | ☆ | ☆ | ☆ | ☆ | |  | ☆ | 8 |
| Wuorela et al,2020 | ☆ | ☆ | ☆ | ☆ | ☆ |  | ☆ | | ☆ | ☆ | 8 |
| Salminen et al,2020 | ☆ | ☆ | ☆ | ☆ | ☆ | ☆ | ☆ | | ☆ | ☆ | 9 |
| Dallmeier et al,2020 | ☆ | ☆ | ☆ | ☆ | ☆ | ☆ | ☆ | | ☆ | ☆ | 9 |
| Wang et al,2019 | ☆ | ☆ | ☆ | ☆ | ☆ | ☆ | ☆ | | ☆ |  | 8 |
| Shi et al,2019 | ☆ | ☆ | ☆ | ☆ | ☆ | ☆ | ☆ | |  | ☆ | 8 |
| Keeble et al,2019 |  | ☆ | ☆ | ☆ | ☆ |  | ☆ | | ☆ | ☆ | 7 |
| Jacobsen et al,2019 | ☆ | ☆ | ☆ | ☆ | ☆ | ☆ | ☆ | |  | ☆ | 8 |
| Zucchelli et al,2018 |  | ☆ | ☆ | ☆ | ☆ | ☆ | ☆ | | ☆ | ☆ | 8 |
| Lee et al,2018 | ☆ | ☆ | ☆ | ☆ | ☆ | ☆ |  | |  | ☆ | 7 |
| Langholz et al,2018 | ☆ | ☆ | ☆ | ☆ | ☆ | ☆ | ☆ | | ☆ | ☆ | 9 |
| Schoufour et al,2017 | ☆ | ☆ | ☆ | ☆ | ☆ | ☆ |  | | ☆ | ☆ | 8 |
| Pereira et al,2017 | ☆ | ☆ | ☆ | ☆ | ☆ | ☆ | ☆ | | ☆ |  | 8 |
| Papachristou et al,2017 |  | ☆ | ☆ | ☆ | ☆ |  | ☆ | |  | ☆ | 6 |
| Hoogendijk et al,2017 | ☆ | ☆ | ☆ | ☆ | ☆ | ☆ | ☆ | | ☆ | ☆ | 9 |
| Turusheva et al,2016 | ☆ | ☆ | ☆ | ☆ | ☆ | ☆ | ☆ | | ☆ |  | 8 |
| Lin et al,2016 | ☆ | ☆ | ☆ | ☆ | ☆ | ☆ | ☆ | |  | ☆ | 8 |
| Hyde et al,2016 |  | ☆ | ☆ | ☆ | ☆ | ☆ | ☆ | | ☆ | ☆ | 8 |
| Díaz de León González et al,2016 | ☆ | ☆ | ☆ | ☆ | ☆ | ☆ |  | |  | ☆ | 7 |
| Bartley et al,2016 | ☆ | ☆ | ☆ | ☆ | ☆ | ☆ |  | | ☆ | ☆ | 8 |
| Jotheeswaran et al,2015 | ☆ | ☆ | ☆ | ☆ | ☆ | ☆ |  | |  | ☆ | 7 |
| Kulmala et al,2014 | ☆ | ☆ | ☆ | ☆ | ☆ | ☆ | ☆ | |  | ☆ | 8 |
| Ravindrarajah et al,2013 | ☆ | ☆ | ☆ | ☆ | ☆ | ☆ |  | |  | ☆ | 7 |
| Garre-Olmo et al,2013 | ☆ | ☆ | ☆ | ☆ | ☆ | ☆ |  | |  | ☆ | 7 |
| Abizanda et al,2013 | ☆ | ☆ | ☆ | ☆ | ☆ | ☆ | ☆ | |  | ☆ | 8 |
| Rockwood et al,2011 | ☆ | ☆ | ☆ | ☆ | ☆ | ☆ | ☆ | | ☆ | ☆ | 9 |
| Graham et al,2009 | ☆ | ☆ | ☆ | ☆ | ☆ | ☆ | ☆ | | ☆ | ☆ | 9 |
| Avila-Funes et al,2009 | ☆ | ☆ | ☆ | ☆ | ☆ | ☆ |  | |  | ☆ | 7 |
| Ensrud et al,2007 | ☆ | ☆ | ☆ | ☆ | ☆ | ☆ | ☆ | | ☆ | ☆ | 9 |
| Woods et al,2005 | ☆ | ☆ | ☆ | ☆ | ☆ | ☆ |  | | ☆ | ☆ | 8 |
| Fried et al,2005 | ☆ | ☆ | ☆ | ☆ | ☆ | ☆ |  | | ☆ | ☆ | 8 |
| Susanto et al,2018 | ☆ | ☆ |  | ☆ | ☆ | ☆ | ☆ | | ☆ | ☆ | 8 |
| Hao et al,2016 | ☆ | ☆ | ☆ | ☆ | ☆ | ☆ |  | |  | ☆ | 7 |
| Theou et al,2012 | ☆ | ☆ | ☆ | ☆ | ☆ | ☆ |  | | ☆ | ☆ | 8 |
| Jacobs et al,2011 | ☆ | ☆ | ☆ | ☆ | ☆ | ☆ | ☆ | | ☆ | ☆ | 9 |
| Lucicesare et al,2010 | ☆ | ☆ | ☆ | ☆ | ☆ | ☆ | ☆ | |  | ☆ | 8 |
| Searle et al,2008 | ☆ | ☆ | ☆ | ☆ | ☆ | ☆ | ☆ | | ☆ | ☆ | 9 |
| Srinonprasert et al,2018 | ☆ | ☆ | ☆ | ☆ |  |  | ☆ | | ☆ | ☆ | 7 |
